# Isonicotinonitrile–benzoic acid (1/1)

**DOI:** 10.1107/S1600536810003442

**Published:** 2010-02-27

**Authors:** Li-Jing Cui, Xin-Yuan Chen

**Affiliations:** a Ordered Matter Science Research Center, College of Chemistry and Chemical Engineering, Southeast University, Nanjing 210096, People’s Republic of China

## Abstract

In the title 1:1 adduct, C_6_H_4_N_2_·C_7_H_6_O_2_, the carboxyl group and its attached phenyl ring are essentially coplanar, being twisted from each other by a dihedral angle of only 2.05 (3)°. In the crystal, the mol­ecules are connected *via* O—H⋯N and C—H⋯O hydrogen bonds, building an *R*_2_^2^(7) ring. Mol­ecules are further linked through π–π inter­actions [centroid–centroid distance of 3.8431 (8) and 3.9094 (8) Å], leading to a one-dimensional chain parallel to the *b* axis.

## Related literature

For related structures, see: Chen *et al.* (2009 ▶); Fu *et al.* (2008 ▶). For hydrogen-bonding motifs, see: Bernstein *et al.* (1995 ▶); Etter *et al.* (1990 ▶). 
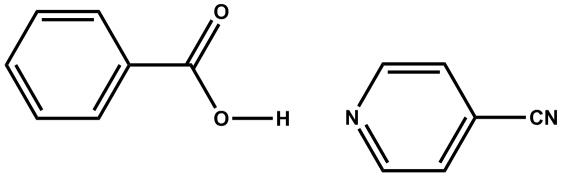


## Experimental

### 

#### Crystal data

C_6_H_4_N_2_·C_7_H_6_O_2_
*M* *_r_* = 226.23
Triclinic, 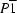

*a* = 7.4274 (15) Å
*b* = 7.7389 (15) Å
*c* = 11.668 (2) Å
α = 85.26 (3)°
β = 76.44 (3)°
γ = 62.79 (2)°
*V* = 579.6 (2) Å^3^
*Z* = 2
Mo *K*α radiation
μ = 0.09 mm^−1^
*T* = 298 K
0.4 × 0.35 × 0.2 mm

#### Data collection

Rigaku Mercury2 diffractometer
Absorption correction: multi-scan (*CrystalClear*; Rigaku, 2005 ▶) *T*_min_ = 0.881, *T*_max_ = 0.940
6025 measured reflections
2646 independent reflections
1346 reflections with *I* > 2σ(*I*)
*R*_int_ = 0.042

#### Refinement

*R*[*F* ^2^ > 2σ(*F* ^2^)] = 0.059
*wR*(*F* ^2^) = 0.154
*S* = 0.96
2646 reflections
154 parameters
H-atom parameters constrained
Δρ_max_ = 0.14 e Å^−3^
Δρ_min_ = −0.18 e Å^−3^

### 

Data collection: *CrystalClear* (Rigaku, 2005 ▶); cell refinement: *CrystalClear*; data reduction: *CrystalClear*; program(s) used to solve structure: *SHELXS97* (Sheldrick, 2008 ▶); program(s) used to refine structure: *SHELXL97* (Sheldrick, 2008 ▶); molecular graphics: *SHELXTL* (Sheldrick, 2008 ▶); software used to prepare material for publication: *SHELXTL*.

## Supplementary Material

Crystal structure: contains datablocks I, global. DOI: 10.1107/S1600536810003442/dn2532sup1.cif

Structure factors: contains datablocks I. DOI: 10.1107/S1600536810003442/dn2532Isup2.hkl

Additional supplementary materials:  crystallographic information; 3D view; checkCIF report

## Figures and Tables

**Table 1:** Hydrogen-bond geometry (Å, °)

*D*—H⋯*A*	*D*—H	H⋯*A*	*D*⋯*A*	*D*—H⋯*A*
O1—H1⋯N1	0.90	1.83	2.726 (2)	176
C8—H8⋯O2	0.93	2.53	3.222 (3)	131

**Fig. 1.**
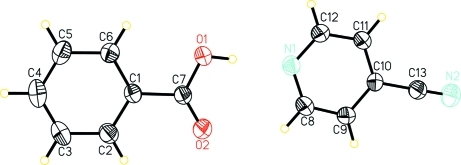
A view of the title compound with the atomic numbering scheme. Displacement ellipsoids were drawn at the 30% probability level.

**Fig. 2.**
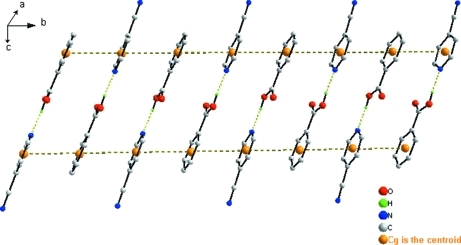
The crystal packing of the title compound, showing the 1D chain. H atoms not involved in hydrogen bonding (dashed line) have been omitted for clarity.

## supplementary crystallographic information

### Comment

Cocrystal attracted more and more attention in recent years for its wide range of applation, for example phase transition dielectric materials and pharmaceutical(Chen, *et al.* 2009; Fu, *et al.* 2008). With the purpose of obtaining cocrystals of isonicotinonitrile, its interaction with various acids has been studied and we have elaborated a serie of new materials with this organic molecule. In this paper, we describe the crystal structure of the title compound, isonicotinonitrile benzoate.

The asymmetric unit is composed of a discrete isonicotinonitrile and benzoic acid molecules (Fig.1). The carboxyl and its parent phenyl ring are essentially coplanar, and only twisted from each other by a dihedral angles of 2.05 (3)°. The two molecules are nearly planar and are only slightly twisted by a dihedral angle of 1.87 (7)° . The molecules were connected *via* O—H···N and C-H···O hydrogen bonds building a R^2^_2_(7) ring (Etter *et al.*, 1990; Bernstein *et al.*, 1995) which play an important role in stabilizing the structural conformation. The molecules units are further linked by weak offset π···π interactions leading to a one-dimensional chain parallel to the *b* axis (Table 2 and Fig. 2).

### Experimental

The commercial isonicotinonitrile and benzoic acid (1/1 mol rate) were dissolved in water/methanol (5:3 v/v) solution. The solvent was slowly evaporated in air affording colourless block-shaped crystals of the title compound suitable for X-ray analysis.

While the permittivity measurement shows that there is no phase transition within the temperature range (from 100 K to 400 K), and the permittivity is 5.9 at 1 MHz at room temperature.

### Refinement

All H atoms attached to C atoms were positioned geometrically and treated as riding, with C—H = 0.93 Å and *U*_iso_(H) = 1.2U_eq_(C) and the H atoms of carboxyl O located in difference Fourier maps and freely refined. In the last stage of refinement they were treated as riding on the O atom, with *U*_ĩso_(H) = 1.5*U_eq_*(O).

### Crystal data

**Table:**

C_6_H_4_N_2_·C_7_H_6_O_2_	*Z* = 2
*M**_r_* = 226.23	*F*(000) = 236
Triclinic, *P*1	*D*_x_ = 1.296 Mg m^−^^3^
Hall symbol: -P 1	Mo *K*α radiation, λ = 0.71073 Å
*a* = 7.4274 (15) Å	Cell parameters from 1346 reflections
*b* = 7.7389 (15) Å	θ = 3.2–27.5°
*c* = 11.668 (2) Å	µ = 0.09 mm^−^^1^
α = 85.26 (3)°	*T* = 298 K
β = 76.44 (3)°	Block, colourless
γ = 62.79 (2)°	0.4 × 0.35 × 0.2 mm
*V* = 579.6 (2) Å^3^	

### Data collection

**Table:**

Rigaku Mercury2 diffractometer	2646 independent reflections
Radiation source: fine-focus sealed tube	1346 reflections with *I* > 2σ(*I*)
graphite	*R*_int_ = 0.042
Detector resolution: 13.6612 pixels mm^-1^	θ_max_ = 27.5°, θ_min_ = 3.2°
ω scans	*h* = −9→9
Absorption correction: multi-scan (*CrystalClear*; Rigaku, 2005)	*k* = −10→10
*T*_min_ = 0.881, *T*_max_ = 0.940	*l* = −15→15
6025 measured reflections	

### Refinement

**Table:**

Refinement on *F*^2^	Primary atom site location: structure-invariant direct methods
Least-squares matrix: full	Secondary atom site location: difference Fourier map
*R*[*F*^2^ > 2σ(*F*^2^)] = 0.059	Hydrogen site location: inferred from neighbouring sites
*wR*(*F*^2^) = 0.154	H-atom parameters constrained
*S* = 0.96	*w* = 1/[σ^2^(*F*_o_^2^) + (0.0699*P*)^2^] where *P* = (*F*_o_^2^ + 2*F*_c_^2^)/3
2646 reflections	(Δ/σ)_max_ < 0.001
154 parameters	Δρ_max_ = 0.14 e Å^−^^3^
0 restraints	Δρ_min_ = −0.18 e Å^−^^3^

### Special details

**Table:**

Geometry. All esds (except the esd in the dihedral angle between two l.s. planes) are estimated using the full covariance matrix. The cell esds are taken into account individually in the estimation of esds in distances, angles and torsion angles; correlations between esds in cell parameters are only used when they are defined by crystal symmetry. An approximate (isotropic) treatment of cell esds is used for estimating esds involving l.s. planes.
Refinement. Refinement of *F*^2^ against ALL reflections. The weighted *R*-factor wR and goodness of fit *S* are based on *F*^2^, conventional *R*-factors *R* are based on *F*, with *F* set to zero for negative *F*^2^. The threshold expression of *F*^2^ > σ(*F*^2^) is used only for calculating *R*-factors(gt) etc. and is not relevant to the choice of reflections for refinement. *R*-factors based on *F*^2^ are statistically about twice as large as those based on *F*, and *R*- factors based on ALL data will be even larger.

### Fractional atomic coordinates and isotropic or equivalent isotropic displacement parameters (Å^2^)

**Table:**

	*x*	*y*	*z*	*U*_iso_*/*U*_eq_	
O1	0.3710 (2)	0.8106 (2)	0.47118 (13)	0.0701 (5)	
H1	0.4865	0.8033	0.4202	0.105*	
O2	0.5897 (2)	0.6595 (2)	0.58797 (14)	0.0824 (5)	
N1	0.7138 (3)	0.8082 (2)	0.32068 (15)	0.0578 (5)	
C1	0.2396 (3)	0.7201 (3)	0.65759 (17)	0.0491 (5)	
C7	0.4176 (3)	0.7263 (3)	0.56976 (18)	0.0529 (5)	
C9	1.0739 (3)	0.7156 (3)	0.29472 (19)	0.0615 (6)	
H9	1.1922	0.6611	0.3257	0.074*	
C6	0.0455 (3)	0.7920 (3)	0.63365 (19)	0.0595 (6)	
H6	0.0224	0.8473	0.5614	0.071*	
C11	0.9010 (3)	0.8656 (3)	0.13992 (18)	0.0584 (6)	
H11	0.9012	0.9140	0.0643	0.070*	
C8	0.8875 (3)	0.7304 (3)	0.35995 (18)	0.0610 (6)	
H8	0.8827	0.6831	0.4359	0.073*	
C10	1.0797 (3)	0.7841 (3)	0.18209 (18)	0.0503 (5)	
C2	0.2716 (3)	0.6398 (3)	0.76576 (18)	0.0616 (6)	
H2	0.4022	0.5920	0.7825	0.074*	
C12	0.7228 (3)	0.8737 (3)	0.21192 (19)	0.0612 (6)	
H12	0.6022	0.9278	0.1832	0.073*	
C13	1.2710 (3)	0.7712 (3)	0.1076 (2)	0.0642 (6)	
C5	−0.1155 (4)	0.7817 (3)	0.7179 (2)	0.0717 (7)	
H5	−0.2463	0.8285	0.7015	0.086*	
N2	1.4213 (3)	0.7615 (3)	0.04740 (19)	0.0934 (8)	
C4	−0.0823 (4)	0.7023 (3)	0.8254 (2)	0.0762 (7)	
H4	−0.1913	0.6979	0.8821	0.091*	
C3	0.1099 (4)	0.6302 (3)	0.8492 (2)	0.0719 (7)	
H3	0.1325	0.5747	0.9215	0.086*	

### Atomic displacement parameters (Å^2^)

**Table:**

	*U*^11^	*U*^22^	*U*^33^	*U*^12^	*U*^13^	*U*^23^
O1	0.0597 (9)	0.0992 (12)	0.0555 (10)	−0.0427 (9)	−0.0132 (7)	0.0230 (8)
O2	0.0527 (10)	0.1130 (13)	0.0769 (12)	−0.0362 (9)	−0.0181 (8)	0.0305 (10)
N1	0.0573 (11)	0.0652 (11)	0.0517 (11)	−0.0313 (9)	−0.0058 (9)	0.0008 (9)
C1	0.0536 (13)	0.0467 (12)	0.0467 (12)	−0.0242 (10)	−0.0075 (10)	0.0021 (9)
C7	0.0512 (13)	0.0548 (13)	0.0512 (13)	−0.0243 (11)	−0.0099 (10)	0.0070 (10)
C9	0.0539 (13)	0.0739 (15)	0.0575 (14)	−0.0289 (11)	−0.0175 (10)	0.0124 (11)
C6	0.0581 (14)	0.0697 (14)	0.0572 (14)	−0.0346 (12)	−0.0131 (11)	0.0046 (11)
C11	0.0569 (13)	0.0664 (14)	0.0519 (13)	−0.0292 (11)	−0.0132 (11)	0.0130 (11)
C8	0.0659 (15)	0.0683 (14)	0.0488 (13)	−0.0324 (12)	−0.0110 (11)	0.0088 (11)
C10	0.0488 (12)	0.0512 (12)	0.0515 (12)	−0.0253 (10)	−0.0071 (9)	0.0027 (9)
C2	0.0623 (14)	0.0644 (14)	0.0547 (14)	−0.0273 (11)	−0.0117 (11)	0.0074 (11)
C12	0.0519 (12)	0.0753 (15)	0.0570 (14)	−0.0292 (11)	−0.0147 (10)	0.0096 (11)
C13	0.0540 (14)	0.0720 (15)	0.0629 (15)	−0.0271 (12)	−0.0123 (12)	0.0101 (11)
C5	0.0562 (14)	0.0832 (17)	0.0800 (18)	−0.0386 (13)	−0.0056 (13)	−0.0037 (13)
N2	0.0625 (13)	0.126 (2)	0.0867 (17)	−0.0462 (14)	−0.0044 (12)	0.0156 (14)
C4	0.0825 (18)	0.0744 (16)	0.0691 (17)	−0.0466 (15)	0.0151 (14)	−0.0085 (13)
C3	0.0852 (18)	0.0718 (16)	0.0497 (14)	−0.0353 (14)	−0.0008 (13)	0.0068 (11)

### Geometric parameters (Å, °)

**Table:**

O1—C7	1.313 (2)	C11—C10	1.376 (3)
O1—H1	0.9025	C11—H11	0.9300
O2—C7	1.205 (2)	C8—H8	0.9300
N1—C12	1.325 (3)	C10—C13	1.446 (3)
N1—C8	1.326 (3)	C2—C3	1.384 (3)
C1—C6	1.377 (3)	C2—H2	0.9300
C1—C2	1.383 (3)	C12—H12	0.9300
C1—C7	1.488 (3)	C13—N2	1.144 (3)
C9—C8	1.373 (3)	C5—C4	1.375 (3)
C9—C10	1.375 (3)	C5—H5	0.9300
C9—H9	0.9300	C4—C3	1.363 (3)
C6—C5	1.388 (3)	C4—H4	0.9300
C6—H6	0.9300	C3—H3	0.9300
C11—C12	1.368 (3)		
			
C7—O1—H1	110.0	C9—C10—C11	119.40 (19)
C12—N1—C8	117.62 (18)	C9—C10—C13	120.88 (19)
C6—C1—C2	119.62 (19)	C11—C10—C13	119.72 (19)
C6—C1—C7	121.69 (18)	C1—C2—C3	120.2 (2)
C2—C1—C7	118.69 (18)	C1—C2—H2	119.9
O2—C7—O1	123.03 (18)	C3—C2—H2	119.9
O2—C7—C1	122.61 (18)	N1—C12—C11	123.1 (2)
O1—C7—C1	114.36 (18)	N1—C12—H12	118.4
C8—C9—C10	117.7 (2)	C11—C12—H12	118.4
C8—C9—H9	121.2	N2—C13—C10	179.1 (3)
C10—C9—H9	121.2	C4—C5—C6	120.2 (2)
C1—C6—C5	119.7 (2)	C4—C5—H5	119.9
C1—C6—H6	120.2	C6—C5—H5	119.9
C5—C6—H6	120.2	C3—C4—C5	120.2 (2)
C12—C11—C10	118.44 (19)	C3—C4—H4	119.9
C12—C11—H11	120.8	C5—C4—H4	119.9
C10—C11—H11	120.8	C4—C3—C2	120.0 (2)
N1—C8—C9	123.7 (2)	C4—C3—H3	120.0
N1—C8—H8	118.2	C2—C3—H3	120.0
C9—C8—H8	118.2		

### Hydrogen-bond geometry (Å, °)

**Table:**

*D*—H···*A*	*D*—H	H···*A*	*D*···*A*	*D*—H···*A*
O1—H1···N1	0.90	1.83	2.726 (2)	176
C8—H8···O2	0.93	2.53	3.222 (3)	131

### Table 2 π–π interactions (Å, °)

Cg1 is the centroid of C1–C6 and Cg2 is the centroid of N1–C12.

**Table:**

	Centroid–centroid	Plane–plane	Offset
Cg1—- Cg2^i^	3.83	3.52	23.2
Cg1—-Cg2^ii^	3.91	3.59	23.3

Symmetry codes: (i) 1-x, 1-y, 1-z; (ii) 1-x, 2-y, 1-z.
